# Remote science at sea with remotely operated vehicles

**DOI:** 10.3389/frobt.2024.1454923

**Published:** 2024-10-18

**Authors:** Zara Mirmalek, Nicole A. Raineault

**Affiliations:** ^1^ NASA Ames Research Center, Moffett Field, CA, United States; ^2^ BAER Institute, Moffett, CA, United States; ^3^ Florida Institute of Oceanography, University of South Florida, St. Petersburg, FL, United States

**Keywords:** remotely operated vehicle, remote science, telepresence, deep-sea, accessibility, ocean exploration, ethnography, marine technology

## Abstract

Conducting sea-going ocean science no longer needs to be limited to the number of berths on a ship given that telecommunications, computing, and networking technologies onboard ships have become familiar mechanisms for expanding scientists’ reach from onshore. The oceanographic community routinely works with remotely operated vehicles (ROVs) and pilots to access real-time video and data from the deep sea, while onboard a ship. The extension of using an ROV and its host vessel’s live-streaming capabilities has been popularized for almost 3 decades as a telepresence technology. Telepresence-enabled vessels with ROVs have been employed for science, education, and outreach, giving a greater number of communities viewing access to ocean science. However, the slower development of technologies and social processes enabling sustained real-time involvement between scientists on-ship and onshore undermines the potential for broader access, which limits the possibility of increasing inclusivity and discoveries through a diversity of knowledge and capabilities. This article reviews ocean scientists’ use of telepresence for ROV-based deep-sea research and funded studies of telepresence capabilities. The authors summarize these studies findings and conditions that lead to defining the use of telepresence-enabled vessels for “remote science at sea.” Authors define remote science at sea as a type of ocean expedition, an additional capability, not a replacement for all practices by which scientists conduct ocean research. Remote science for ocean research is an expedition at-sea directed by a distributed science team working together from at least two locations (on-ship and onshore) to complete their science objectives for which primary data is acquired by robotic technologies, with connectivity supported by a high-bandwidth satellite and the telepresence-enabled ship’s technologies to support the science team actively engaged before, during, and after dives across worksites. The growth of productive ocean expeditions with remote science is met with social, technical, and logistical challenges that impede the ability of remote scientists to succeed. In this article, authors review telepresence-enabled ocean science, define and situate the adjoined model of remote science at sea, and some infrastructural, technological and social considerations for conducting and further developing remote science at sea.

## 1 Introduction

Scientific ocean studies routinely use telecommunication tools for near-real-time (low-latency) communication transmissions between scientists and operators on a ship with robots in the sea (e.g., remotely operated vehicles, autonomous underwater vehicles, gliders, etc.). Yet, the extension of what is fundamentally a distributed work model (i.e., workgroups in different locations co-operating in the production of a shared goal) to maintain ongoing real-time connectivity with scientists onshore, such that their geographic remoteness to the ship does not exclude them from active participation in science directions and decision-making, has been slow to develop. Telecommunications, computing, and networking technologies onboard ships have become familiar mechanisms for expanding scientists’ reach from onshore. The deep-sea oceanographic community routinely works with remotely operated vehicles (ROVs) and ROV pilots to access real-time live-stream video from the deep sea, collecting data while onboard a ship. Given the technological capabilities of ocean robots and available shipboard and onshore telecommunication technologies, what might the ocean science community consider as some of the inhibitors to increasing the size of ocean expedition teams via remote science at sea? Remote science at sea is defined here as an expedition at-sea directed by a distributed science team working together from at least two locations (on-ship and onshore) to complete their science objectives for which primary data is digital media (video, audio, and sensor data) or material (e.g., samples) acquired by robotic technologies, with connectivity supported by a high-bandwidth satellite and the telepresence-enabled ship’s telecommunication, computing and networking technologies to support the science team actively engaged in maintaining communication and situational awareness before, during, and after dives across worksites.

Authors put forward a brief timeline to situate the historical and social development of remote science at sea through telepresence tools enabling ocean science, education, and outreach. The selected markers highlight events that made significant structural and cultural impressions, ultimately, in the work landscape for ocean science with ROVs. One marker for the start of this timeline is in 1872 when the first state-sponsored expedition (Great Britain) dedicated to conducting ocean science set out aboard the HMS *Challenger* (Burstyn, 1972). Indeed, in preceding centuries, individuals [e.g., Posidonius (who preceded Aristotle) to Robert Boyle], and professions (e.g., sailors, tradespersons), were building marine science knowledge including observations on tides, salinity, and sound (Deacon, 1971). The *Challenger*’s four-year expedition carried six scientists (with 243 crew) and a science lab onboard, completing 362 sampling stations and 133 dredges. This is a marker against which to consider developments in the field of ocean science and use of robots to extend and increase scientists’ reach. The next marker is in 1880 for the concept of visual telegraphy, “seeing by telegraph,” worked on by numerous persons across continents (*Visual Telegraphy*, 1880), including Alexander Graham Bell, a speech therapist who understood the importance of face-to-face communication, remembered as the inventor of the telephone and founder of Bell Labs. Over half-a-century later in 1942 telepresence appears in popular literature, in a science fiction story about an engineer who designs and remotely operates teams of devices as extensions of his hands (MacDonald, 1942). The story is notable for its description of telepresence extending human reach and for the author, an engineer and science-fiction writer often credited with shaping people’s imaginations on social and technological futures (Robert A. Heinlein wrote under the pseudonym MacDonald). In 1980, Dr. Marvin Minsky, a designer of robotic arms and co-founder of the Artificial Intelligence Lab at the Massachusetts Institute of Technology, declared “telepresence” a new term for teleoperator, a person using a robotic arm to work in an environment that is harmful to human physiology (Minsky, 1980). His use of the term telepresence, he stated, was intended to elevate “the importance of high-quality sensory feedback; ” instruments used by tele-operators should “feel and work so much like our own hands that we won’t notice any significant difference. Using instruments, you can “work” in another room, in another city, in another country, or on another planet.” A few years later, telepresence appeared in front of a global audience through the work of Dr. Robert Ballard and his description of its use in the 1985 discovery of the wreck of the *Titanic* (Ballard, 1985; Chamberland, 1987).

The idea for a connected ship-and-shore research workspace originated in the early 1980’s. The concept for the first scientific ROV, *Jason,* developed at Woods Hole Oceanographic Institution (WHOI) in 1982, included a satellite link for shore-based control and data processing centers (Ballard, 1982). The long-term goal was to enable a network with scientists and operators onshore to access and control vehicles at sea for a cost-effective alternative to human-occupied vehicles (HOVs) and more time for benthic research (Ballard, 1982; Ballard, 1993). Initial ship-to-shore connectivity was provided through MARISAT, the world’s first satellite communications system for ships, launched in 1976 for the shipping industry and superseded by Inmarsat in 1982, providing international and nonmilitary satellite service to a wide variety of vessels and at-sea platforms (Ilcev, 2019). Still, communication was limited to email exchanges or short calls on a satellite phone and research ships remained largely a self-contained environment. Researchers were trained and expected to be almost entirely cut off from shore during expeditions. Since 2010, a growing number of ocean exploration research vessels began utilizing seagoing satellite dishes with high-bandwidth capabilities to stream ROV video to shore, often for outreach (Coleman et al., 2014), but also to add experts for ROV dive leadership (see Section 2.2). In the past several years, with the proliferation of Low-Earth Orbiting satellite systems, the newest cohort of researchers are experiencing a highly connected ship-and-shore environment on a growing number of research vessels that is similar to their expectations for continuous service and ability for data, video, and audio streaming (NOAA Office of Ocean Exploration and Research, 2020). Oceanographic researchers are now in an era where most vessels are equipped with marine satellites, enabling connectivity and sharing of the large datasets from robotic platforms to shore in near-real time. Authors draw from these events in the last two decades (2005–2022) to review telepresence-enabled ocean science studies and situate remote science at sea.

Without increasing the personnel capacity of research vessels, remote science offers expanding capacity that supports accessibility for more persons interested in ocean science, careers in the new blue economy, and persons with varying backgrounds and expertise. For many reasons, including human physical needs, family care, schedules, health needs, and social networks, vessels are not accessible to everyone, which limits who can be involved in at-sea research. In addition, the size of research vessels being added to the fleet in the United States is shrinking, offering fewer bunks for researchers, and fewer ships capable of reaching the deep sea globally (National Research Council, 2015). Research vessels capable of deploying deep-sea robotic assets are also over-subscribed for funded research projects, creating a backlog of researchers waiting for access to the sea (National Research Council, 2004; National Research Council, 2015). Remote science adds to the solutions by offering researchers conducting science in the same location or with compatible research goals, shared vessel and ROV time bolstered by additional team members leading from shore. Furthermore, researchers who understand remote science technologies can choose to plan their expeditions and write proposals to include and support leadership from shore. Funding considerations are discussed later in this article.

Telepresence tools create a communications connection between ROVs and people on-ship and onshore, which is now familiar to many. However, broadening and further developing telepresence to support remote science at sea is not a simple progression. As with any technology, development and adoption is shaped by cultural values and rewards. Does a community value the use of a technology and thus reward the user and popularize the technology, or is it negatively considered, thus stigmatizing those that use it? For ocean scientists, the activity of seagoing is a long-standing tradition and valued feature of their work. The community recognizes sea-going scientists with numerous rewards, from professional recognition and advancement, to the tradition of earning the right to specific certificates (sometimes memorialized as tattoos) symbolizing having sailed in particular areas or distances. Remote science has been routine for space exploration as space scientists remain on Earth while collecting data off-Earth with remote sensing tools and robots. It has not been routine for ocean scientists. And yet, human-robot teaming has been part of ocean research expeditions for decades and has yielded measurable outcomes including scientific breakthroughs, new data and specimens for repositories, research publications, new species discoveries, and content for science communication. Between 2010–2016, a collection of nineteen scientific discoveries and new research techniques were made using telepresence-enabled ocean science and published in a special issue of an ocean science journal, which also highlighted some different modes and uses of telepresence, such as uses of communication tools for experts onshore to “drop-in” on-ship (their role has been referred to by a few names such as “Doctors on Call,” “Doctors on Duty,” and “Scientists Ashore”) and inclusion of a co-located onshore team (Raineault et al., 2018). This example is not the rule–it is still rare for scientists to record their use of telepresence or provide methodical details of its use in their publications. The acknowledgement of the vessel, expedition number and possibly a funding source in a manuscript does not denote telepresence was used, which means that one either needs to already know it was a condition on an expedition or look to other sources to learn whether telepresence was used. Today, we must consider, almost two decades since the first studies of telepresence-enabled science at-sea demonstrated their effectiveness, the possible conditions that limit and those that promote widespread understanding, adoption, and acceptance of remote technologies for conducting deep-sea research.

## 2 Broadening real-time reach at sea

### 2.1 First large-scale telepresence-enabled expedition, Lost City (2005)

In 2005, the Lost City expedition led by Dr. Deborah Kelley was the first to test a large-scale telepresence operational model for both science and outreach (Kelley, 2005; Kelley et al., 2007; Kelley et al., 2007). ROVs *Hercules* and *Argus* were deployed from the R/V *Ronald H. Brown* at the Lost City hydrothermal vent field at the mid-Atlantic Ridge. Dr. Kelley’s shoreside team of 21 scientists, graduate students, and undergraduates led a 24/7 expedition for nine-days from the University of Washington’s Science Command Center (Kelley et al., 2007).

Telepresence technology was set up by a team of communication network experts from the University of Rhode Island’s Graduate School of Oceanography. They installed an intercom panel and several Tandberg decoders for video streams at the Science Command Center, taking advantage of the Internet2 high-bandwidth capabilities. Internet2 is a company that provides a secure, high-speed network, among other services, for research and education institutions. Transmissions between ship and shore included video, voice, and data with a latency of ∼1.5 s (Kelley et al., 2007). Over the course of the expedition, there were 20 h of live broadcasts (forty sessions, each 30-min in duration) from the vessel to University of Washington (UW) with students enabled to interact in real-time with the shipboard scientists (Kelley et al., 2007). The live broadcasts included a variety of sites from K-12 classrooms, museums, aquariums, and science centers throughout the U.S. through Immersion Presents, Boys and Girls Clubs of America, and the Jason Foundation for Education (Ballard, 2005). The expedition collected high-definition video, still imagery, and rock samples that improved the understanding of the vent field (Denny et al., 2016; Kelley et al., 2007).

Lost City expedition’s publications and subsequent projects that included telepresence by some of the science participants (e.g., R. Ballard; D. Butterfield; A. Caporaso; D. Coleman; P. Girguis; J. Karson; C. Martinez, B. Phillips; C. Roman; T. Shank) suggest the telepresence capability was useful. Indeed, authors suggest a social network analysis using the Lost City expedition participant list may yield some findings on best practices, technology with the quickest or slowest uptake with the professional community in a two-decade timescale. The telepresence technology passed its first test, showing an ability to support scientists working at sea from onshore. However, for uptake as a valued tool by a professional community for whom physical presence at sea has high value there would need to be continued use and an elevation of details on the “how.” That is, how does a team that is accustomed, through training and habit, to being co-located (on a ship) work together using telepresence? Answers to this question, not posed in the project itself, would allow for systematic progress, including scientific testing, of telepresence-enabled ocean research for broader use. Costs of a telepresence-enabled expedition also need to be more widely known, for science planning purposes and funding requests. Laura Ruth’s “Gambling at Sea” article on the costs of deep-sea research includes a comment on the use of shore-based scientists, ROVs, and satellite transmissions from a scientist on the Lost City expedition who said, “that [it] could become a cost-saving tool in the future, but it is not yet sufficiently cost-effective” (Ruth, 2006). No additional details are provided to evaluate that statement.

### 2.2 Telepresence-enabled ocean exploration (2010–2022)

In 2010, E/V *Nautilus* and National Oceanic and Atmospheric Administration (NOAA) Ship *Okeanos Explorer*, two U.S.-funded vessels for scientific exploration of the ocean began operating with telepresence capabilities to support scientific, education and outreach goals of the U.S. Strategy for Ocean Exploration (Martinez and Keener-Chavis, 2006; President’s Panel for Ocean Exploration, 2000). By 2013 the use of telepresence by the R/V *Falkor*, R/V *Thompson*, and R/V *Atlantis*, supported occasional educational and outreach activities (Coleman et al., 2014). The University of Rhode Island’s Inner Space Center (ISC) was established to provide the telepresence technologies and a facility to connect vessels operating at-sea with shore (Wojtas, 2009). The ISC is comprised of a trained technical staff to operate video production and broadcast systems, ship-to-shore telecommunications equipment, real-time data processing and visualization systems, in a space that mimics the shipboard ROV control room environment. Acting as the distribution hub for streamed video, the ISC redistributes video to other Internet2-equipped “Exploration Command Centers” (ECCs) at research and education institutions or the internet. The “Mission Control” part of the ISC was designed to host large teams of shoreside scientists and includes a large projector wall that can display the ROV or other shipboard video feeds and rows of desks with computers and monitors that can pull from data synced to shore or display shared shipboard displays for situational awareness. Communications equipment common to the vessels and shore (as well as ECCs), allow shoreside and shipboard scientists and operators to have real-time discussions. The ISC can also record and archive video and other ROV data.

The annual summaries (2011–2023) in the Oceanography Society’s “New Frontiers in Ocean Exploration” supplement include highlights of the use of telepresence and changes in technologies and practices. Initially, the ship and shore-based hub that redistributed video and data from the vessels at sea were required to have video production and broadcast systems for transmission of and receipt of video, audio and data from the vessel. The ship required a specialized sea-going satellite dish and high-bandwidth service. Once video and data were received at the Inner Space Center, which was the hub, it was distributed over Internet2 connection to other ECCs located at several other universities and institutions. Experts at these locations had similar communications systems to the vessel and could talk to the on-ship watchstanders to provide support.

In 2011, live video from a vessel and ROV and audio from operators in the ROV control room were streamed over standard home internet connection, expanding viewership. Standard internet streaming has increased latencies (time-delay). It was primarily intended for education and outreach purposes, with the use of ECCs preferred for research involvement. However, scientists who were not located at an ECC were still able to view a livestream and call a phone line on the vessel to provide input as needed. This model for involvement was referred to as “Doctors on Call” and was mainly used by shipboard teams to call in support from shore as needed when a new discovery was encountered outside the shipboard team’s expertise. Both parties would need to work out if and how latency was affecting what they were seeing in the live stream. Over time the interested network of “Scientists Ashore” who registered to view and participate via text-based chat with watchstanders aboard the exploration vessels grew to over 200 per expedition season (Ballard, 2019). With only 3 science watchstanders aboard the *Okeanos Explorer*, there was heavier reliance on the “Doctors-on-duty” located at ECCs to lead and narrate dives (Martinez et al., 2012). Improved streaming capabilities over standard internet and chat-style communications with watchstanders aboard the vessel increased the ability of researchers to be involved via telepresence (e.g., Martinez et al., 2012; Cantwell et al., 2020).

Improvements in technologies and awareness allowed for a growing number of scientists to engage in telepresence-enabled exploration from their home institutions. Voice-over IP (VOIP) could be used to conference scientists into the shipboard watchstanding communications system, allowing them to lead dives from non-ECC locations, with an understood 5-s video latency over standard internet or 2-s over Internet2 (Kennedy et al., 2016; 2020; Delgado et al., 2018). Other improvements that reduced the cost of streaming and viewing ROV video, including cloud services, meant streams could be made accessible to anyone, without incurring additional costs (Peters et al., 2019; Russell et al., 2019). At the same time, improvements in video data compression, along with greater stability of satellite and internet services over this period reduced concerns about lower quality video, latencies, and reduced data availability for shore-based participants. Improved network controls through new firewall technologies improved cybersecurity and the management of data and workflows for multiple uses of the satellite (e.g., live streaming, educational programming, data transfers) (Coleman et al., 2023).

Technology developments and work practices used for operating and maintaining vehicles in space exploration have also opened opportunities for vehicles in ocean teleoperations—whereby engineers or specialists can assist in preparation, troubleshooting, and operations of ocean robots and instrumentation from shore. In 2012, remote robotic operations and telepresence-enabled ocean science were tested using the autonomous underwater vehicle (AUV) *Sentry* operated from the NOAA vessel *Okeanos Explorer* for 20 days at three sites off the U.S. east coast (Kaiser et al., 2012). One of their objectives was to apply and test the practice of remote engineering and remote data processing, which required the cooperation of the operations and science team aboard the vessel and a co-located team of scientists and engineers onshore. Deemed a success, the remote participants allowed an adequate depth of expertise across various oceanographic systems (e.g., AUVs, ROVs, etc.), sensors, and science objectives to allow a multi-robot, multi-disciplinary expedition to be conducted on a vessel that would not have space for all the necessary team members (Kaiser et al., 2012). Since 2016 the *Okeanos Explorer* allows mapping operations to be run by persons on-shore, including giving them the ability to control acquisition computers and process data (Lobecker et al., 2017). In 2021, *Nautilus* demonstrated remote engineering and operation potential using the hybrid ROV *Nereid Under-Ice* vehicle (hROV *NUI*) with operators located aboard the vessel and at WHOI (Dalpe et al., 2022).

While the use of telepresence for science support aboard exploration vessels first stemmed to address the need for expert assistance in the event of a discovery outside of the shipboard personnel’s expertise (e.g., Sowers and Hoy, 2020), it has allowed a greater number of scientists to utilize a vessel than the limited berthing would allow (Martinez et al., 2012). It is a capability with growth potential for the use of multiple robotic platforms or shipboard instruments that require distributed technical teams, whether they cannot be accommodated aboard the vessel due to space limitations, funding, conflicting institutional schedules or responsibilities.

### 2.3 Telepresence-enabled expeditions for seafaring ocean science

Telepresence-enabled expeditions were first set-up as an educational tool for K-12 students. Founded in 1989, the JASON project was created by Dr. Robert Ballard to bring students to sea virtually (Bazler et al., 1993). During and since the aforementioned Lost City Expedition, some ocean scientists began utilizing the telepresence-enabled vessels as an educational and outreach tool in the manner of live-streaming short presentations and two-way question and answer sessions with not only K-12, but also with undergraduate students.

Since the early 2010’s, the exploration vessel’s robust education and outreach component caught the attention of researchers and the public including live streams of the ROV video and audio from researchers (primarily aboard the vessels). Since 2010, an “Educator at Sea” watchstanding role aboard the *Nautilus* was offered to a trained educator to narrate the sea-faring activities and to act as a liaison between the operational and scientific watchstanders and the public onshore. Live streaming to YouTube starting in 2012 also helped popularize the deep-sea video feeds from the exploration vessels (e.g., Russell et al., 2019). Point-to-point broadcasts connect classrooms, aquariums, science centers, and other venues with persons aboard telepresence-enabled ships, sharing information and videos or images and answering questions live. The interest and growth in outreach from vessels expanded to other research vessels, including six aided by “Mobile Telepresence Units” provided and supported by the ISC in 2019 (Peters et al., 2019). Scientists used the model to fulfill broader impacts required by funding agencies for their research.

#### 2.3.1 TREET (2014)

In 2013, the National Science Foundation (NSF) funded a multi-disciplinary study on the use of telepresence for expanding undergraduate learning in ocean science, “Transforming Remotely Conducted Research through Ethnography, Education and Rapidly Evolving Technologies” (TREET). The study proposed “to make important inroads into the mechanisms by which remote human-robotic interactions can be utilized to transform the future of research and how these same systems can be leveraged to advance the research experiences of Early Career Scientists (ECS) and students.” It would accomplish this by investigating the efficacy of using a telepresence setup–a telepresence-enabled vessel, real-time video feeds from two ROVs, a designated onshore facility, an ECC–to train undergraduates students and for Early Career Scientists to increase their opportunities to collect science data (Bell, 2015; Bell et al., 2016; German et al., 2014; Pallant et al., 2016; Stephens et al., 2016).

TREET’s multidisciplinary scope of study was represented by one to several scientists: ocean science and telepresence were represented by P.I. Dr. Christopher German (WHOI) and several other ocean scientists from Woods Hole Oceanographic Institution (WHOI), Monterey Bay Aquarium Research Institute (MBARI), University of Rhode Island (URI), and Ocean Exploration Trust (OET), education was represented by Dr. Amy Pallant and her team (Concord Consortium), and work ethnography was represented by Dr. Zara Mirmalek (Harvard). Additionally, expertise in formulating the ocean science plan with ROVs, telepresence, and education components was provided by Dr. Katy Croff Bell (OET) and Dr. Kanna Rajan (MBARI). The TREET project’s six ECS confirmed their participation during the proposal stage and their eight undergraduates were recruited in fall 2013 after the project was funded.

The study’s focus on telepresence engagement for scientists on shore to robustly participate in directing science plans on-ship was premised by Dr. Mirmalek and Dr. Rajan’s work in remote operations with robots for space exploration. Dr. Mirmalek conducted basic and applied research among scientists co-located at NASA’s Jet Propulsion Laboratory (JPL) in Pasadena, California conducting planetary science and exploration with two remotely operated robots on Mars (Mirmalek, 2020). JPL, a long-standing facility supporting remote robotic operations off-Earth, was used by a group of space and instrument scientists (Squyres et al., 2003; Squyres, 2005). Dr. Rajan’s workgroup from NASA Ames Research Center provided and operated an activity planning tool used on the mission (Bresina et al., 2005). Work ethnography was initially included in the scoping of the TREET project by Dr. Kanna Rajan, whose expertise includes development and adoption of remote and autonomous vehicles for planetary and ocean sciences (Bellingham and Rajan, 2007). Ethnography is the study of meaning-making within a community, which in the case of work ethnography refers to communities brought together by institutional goals in and for production. Meaning-making is learned and shared through habits, values, social norms, practices, assumptions, and languages, that members use explicitly and implicitly to make sense of their activities and to guide behavior and relationships. Dr. Mirmalek’s work ethnography has specifically focused on scientific knowledge production and technology adoption among science communities of practice that include human-robot teams in outer space (Mars, Moon) and in the deep-sea (e.g., Mirmalek, 2017; 2020; 2024; Mirmalek et al., 2021; Mirmalek et al., 2024).

TREET’s telepresence-enabled expedition was scheduled for 14 days of ship time in the southeast Caribbean onboard the E/V *Nautilus* sponsored by a NOAA Ocean Exploration and Research grant to the Ocean Exploration Trust. Fourteen days of facility use and digital communication support were provided at the ISC, and one week of facility use for an ECC was provided at WHOI. During the expedition, the TREET team was to be primarily co-located at ISC for two weeks with a subset relocating to WHOI (thus expanding the distribution to two onshore locations); and one scientist would be on-board to facilitate the ocean science plan directed from shore.

Of TREET’s science team, most were experienced in sea-going ocean science but only a few had experiences with telepresence-enabled science. TREET’s P.I. Dr. German and one of the project’s two expert mentors, Dr. Cindy Van Dover, had briefly employed telepresence during an Autonomous Underwater Vehicle (AUV) exploration of Blake Ridge resulting in the discovery of five new seeps (Brothers et al., 2013; Kintisch, 2013; Wagner et al., 2013). Dr. Steven Carey, the second expert mentor, had experience with telepresence-enabled expeditions and was set to be the primary on-board TREET lead scientist, a role which was needed to be held by an experienced scientist able to be “agnostic” across science objectives.

Preparing students and the ECSs for TREET’s fall 2014 cruise and the use of telepresence for ocean science and education began with a 13-week seminar in the winter and spring of 2014. The seminar was conducted via video conferencing, led by Dr. Pallant and her team (Cynthia McIntyre and Lyn Stephens) from the Concord Consortium, an organization with the mission “To innovate and inspire equitable, large-scale improvements in STEM teaching and learning through technology.” Their experience with online learning for STEM educators and students enabled the TREET project to use a framework that provided a stable communication forum, schedule, and education objectives during their evaluation on the use of telepresence.

The purpose of the TREET’s pre-expedition seminar for the entire TREET team was twofold: 1) to build some face-to-face familiarity and communication interactions, and 2) to learn about sea-going ocean science, and ECSs’ specific science objectives. All TREET team members were required to attend the entire seminar series, with their video camera enabled. The scheduled series was supported with content stored in a password-protected webpage, including seminar meeting information, pictures and profiles of all the TREET participants, and recordings of meetings. In each meeting, one to two ECS presented on their research background and science objectives for the TREET cruise, and students presented their interests and questions, both general and specific to their ideas on working with their ECS during the cruise. Post-expedition, in the following spring (April 2015), the team convened for five video-enabled meetings to review the ECSs’ ocean science research.

TREET’s expedition was carried out over 2-weeks across the end of September and start of October 2014, with seventeen completed dives. P.I. German, from onshore, maintained a close-watch and constant decision-making on meeting science objectives for all the ECSs and students. Of the twenty-one defined objectives, nineteen were completed. TREET team members onshore spent the first week co-located at URI, utilizing the Inner Space Center for telepresence-enabled education, ocean science, and outreach. A subset of the team then relocated and participated from an ECC at WHOI. The distributed teams onshore were in the same time zone and were less than one-hour drive apart. These factors were considered as a matter of communication scheduling, and in the event the team needed to quickly co-locate at URI. ECSs onshore were able to instruct and complete science research (Mittelstaedt and Smart, 2018), as well as those who were onboard (Michel et al., 2015; Michel et al., 2018).

Overall, the TREET evaluation on the use of telepresence for undergraduate education was positive (McIntyre and Pallant, 2016; Pallant et al., 2016; Stephens et al., 2016). Students were enabled to lead from shore, with synchronized leadership cooperation between the chief scientist and ECS onshore and on-ship. Nine of the completed objectives were carried out by students. ECS Dr. Masako Tominaga’s students Laney Hart and Carly Scott presented their completed research–a high-resolution geological characterization of the inner crater of the Kick’em Jenny submarine volcano in the Caribbean–at the American Geophysical Union Meeting in 2015 (Hart et al., 2015).

TREET’s telepresence-enabled outreach was also completed. Continuing the use of OET’s setup onboard the *Nautilus,* the Inner Space Center’s connectivity allowed for scientists to engage in outreach communication, e.g., live commentary and discussions led by the Science Educator (on-ship), with participation from scientists onshore. Indeed, some students discovered their interests lay more in the field of science communication and were enabled to participate as such. The onshore facility also supported a scientist’s meeting with their class of undergraduate students.

TREET’s data on the development of work practices for telepresence-enabled ocean science among ECS onshore had to be marked as inconclusive due to unexpected alterations to onshore conditions. The planned number of ECS scientists co-located onshore was scoped for six but was carried out by two. Ethnographic data collection was not interrupted by this change but had to be re-focused with the possibility of understanding and providing analysis for the community of practice for future considerations. The gradual decline in onshore ECS participation is summarized as follows: The planned number of scientists onshore affecting dives was nine (one chief scientist, one senior scientist expert mentor, one expert on software and robotics for remote science and exploration, and six ECS). The first reduction was only one, but it was a significant qualitative loss of expertise in remote robot operations. Next, two onshore ECS were re-assigned to the on-ship count of TREET scientists, thus changing the balance from one (senior) scientist on-ship to three and reducing the number of scientists onshore. The number of onshore ECSs then dropped from four to two, after another one joined the on-ship group right before the ship left dock and one departed the onshore facility just a few days after the start of the cruise. As such, the actual distribution of the team across sites no longer met the planned study condition. The social scientist, also onshore, responsible for ethnographic data collection on patterns and habits, did not have enough of a representative ECS community. Her field research among the ECS onshore was further narrowed by a project condition that necessitated joining the group (one ECS, three students, chief scientist) that relocated from the Inner Space Center.

Analysis from TREET’s ethnographic data collection on work practice, discourse, human-technology relationships (Mirmalek, 2013) carried forward along several temporal lines. In fall 2013, prior to the TREET expedition, Dr. Mirmalek began collecting ethnographic data onboard the *Nautilus* among scientists and operators on a science expedition conducting ROV exploration of the Kahouanne seamounts, southeast of Montserrat in the northern Lesser Antilles (Carey et al., 2019). Semi-structured interviews were conducted with the TREET scientists prior to cruise and some post-cruise; post-cruise student interviews were conducted by the education team. Some of the data analysis was applied to communication protocols required between distributed teams to maintain context awareness across ship and shore sites. Following the completion of TREET, some analysis was used to set up a small remote operations room at WHOI that P.I. German utilized in a subsequent telepresence-focused project (see section 2.3.2) for which he was the Co-P.I. with P.I. Dr. Darlene Lim (Lim, 2019). As well, although the number of ECSs onshore during the TREET cruise was too small to support findings on patterns and habits that would be significant to the community of practice, the experience and analysis yielded were drawn forward to enable a future project, SUBSEA (Lim et al., 2021). Some of the analysis from TREET that would later be used for scoping conditions for the NASA and NOAA jointly funded study SUBSEA included requiring the science team’s adherence to technical and social protocols (an agreement to place value on these conditions), which if breached would invalidate some proposed experiments.

Advancing focus on ECS training and employing telepresence for ocean science and outreach, following TREET, continued with Dr. Van Dover’s (2017) NSF-funded project “EAGER: Developing At-Sea and Telepresence-Led Deep-Submergence Science Leadership.” She was one of the TREET project’s two senior science experts and one of the two scientists who initially scoped it, drawing it together from an earlier project with Dr. German (see Blake Ridge described earlier, Kintisch, 2013). Dr. Van Dover’s experience extends from ocean science (Van Dover, 1996; 2000) to piloting HOV *Alvin* (Van Dover, 1997) to telepresence-enabled expedition planning as an area of expertise. The EAGER project’s 11-day cruise included use of Research Vessel *Atlantis,* the URI’s Inner Space Center, a human-occupied vehicle (*Alvin*), and an autonomous underwater vehicle (*Sentry).* Dr. Van Dover reported (2017) that: “Two dozen ECS participated in person and 56 individuals signed up to tune in remotely. New research on methane seeps was carried out with *Alvin*, *Sentry*, and ship-deployed, standard CTD and hydrographic wire tools on the Mid-Atlantic slope south of Woods Hole MA, including geological, chemical, oceanographic, and biologic sampling and characterizations (McVeigh et al., 2018; Netburn et al., 2018). Telepresence was used for both scientific purposes and outreach between ship and shore, with an aim to assess its effectiveness and explore ECS-driven modes of using it. Outreach was multi-pronged, with a variety of social media avenues, live museum interviews, traditional media spotlights and public web streaming of shipboard video.” As well, some of the ECSs completed the project with a favorable disposition on adoption of telepresence for ocean science (Marlow et al., 2017). The project’s full report (Van Dover et al., 2017) demonstrates notable accounting on telepresence-enabled outreach and education.

#### 2.3.2 SUBSEA project: two telepresence-enabled expeditions with different science operations requirements

In 2017, NASA and NOAA jointly funded a project titled: “Systematic Underwater Biogeochemical Science and Exploration Analog” (SUBSEA) that included two research expeditions, over a two year-period (Lim et al., 2021). SUBSEA project was a multidisciplinary combination that was specifically called for by the NASA funding component, a Planetary Science and Technology from Analog Research (PSTAR), which awards interdisciplinary integrated field experiments drawing together three pillars of Science, Science Operations, and Technology. SUBSEA’s P.I. Dr. Darlene Lim (NASA Ames Research Center) brought experience across the pillars, with operations led in the field of space analogue missions, geobiology, and technology development (e.g., Lim et al., 2011; Lim et al., 2019). Co-P.I. Dr. Christopher German acted as chief scientist working with six to ten co-lead scientists. SUBSEA’s ocean science objectives focused on venting fluids at isolated seamounts and spreading ridges in the Pacific Ocean as analog environments to putative volcanically-hosted hydrothermal systems on other Ocean Worlds. Science Operations studied and tested how the ship and shore architectures, distributed teams, communication, and telepresence environment would fare as an analog environment for developing human space exploration. Dr. Mirmalek joined to lead the Operations research (with Dr. Matthew Miller, Georgia Institute of Technology). The technology pillar, led by Dr. Matthew Deans (NASA Ames) and his team (Tamar Cohen and Dr. David Lees, NASA Ames), was a suite of web tools called xGDS (Exploration Ground Data System) that provided scientists with temporally synchronized and mapped location of observation notes, instrument data, photos, video, samples and other data (Cohen et al., 2020). Expedition planning, human-robot teaming, and enabling on-board cooperation with onshore direction from ocean scientists was led by Dr. Nicole Raineault (Ocean Exploration Trust).

Planning for 2018 and 2019 expeditions began in fall 2017. The SUBSEA team carried out two in-person workshops (fifteen to twenty scientists and technology specialists) to ascertain and share knowledge and goals. To provide a compromise between the traditional ocean science at-sea experience and allow for a comparative evaluation on telepresence capabilities, Dr. Mirmalek proposed changing communication conditions between the two cruises. For “Cruise A” in 2018, the experienced ocean scientists determined who would be located on-ship and onshore and how often they would communicate and through which mediums (e.g., email, text, phone, chat field on the software interface used on-ship and onshore). “Cruise B” in 2019 would draw from research during Cruise A on communication, interaction, and work practices that would be used to set up Cruise B protocols for required communication conditions.

SUBSEA Cruise A: The 2018 expedition utilized telepresence-enabled ocean science and outreach at the Kamaʻehuakanaloa (formerly Loi’hi) Seamount off the southeast coast of Hawai’i. Between August 21 and September 12, fourteen ROV dives primarily led by scientists on-board the E/V *Nautilus* gathered new data and samples on the active venting processes (Hand and German, 2018; Huber et al., 2022; Milesi et al., 2023; Soule et al., 2019). Onshore science team members were located at the URI’s Inner Space Center and in a scientist’s lab space at WHOI that had been customized for telepresence participation (drawing from TREET analysis). The Technology team was onshore at the ISC. Operations scientists were distributed between on-ship and onshore (Miller et al., 2019). The traditional practices of a chief scientist on-board were followed, and ocean scientists were allowed to self-direct communication between ship and shore. Operations included only two parameters: 1) a daily teleconference between ship and shore team (approximately 30 minutes); and 2) completing a daily four-question survey on unscheduled communication between individuals on-ship and shore. The daily teleconference calls were recorded and analyzed by Operations for setting up communication protocols for Cruise B. The four-question survey tracked communication mediums, frequency, purpose, and criticality level. The survey results were used to evaluate whether the team of scientists’ work objectives would be hampered by restricting these habits in Cruise B. The results indicated they would not be hampered.

SUBSEA Cruise B: The 2019 expedition operated under remote science conditions at the SeaCliff hydrothermal vent field at Gorda Ridge off the coast of Oregon. Between May 22 and June 9, seven ROV dives were led by scientists onshore at the ISC. During Cruise B, the chief scientist was not onboard and, instead, was asked to direct and lead co-operation from onshore. None of the lead scientists directing data collection were allowed on-board. SUBSEA team members and crew onboard were instructed to support science directions from shore. Although this restricted access was not easy to enforce, it was necessary based on lessons Operations Lead Dr. Mirmalek had learned from the TREET study. Some lead scientists had members of the lab onboard to handle specialized samples and instrumentation. The Technology team was onshore supporting the use of xGDS, and the Operations scientists were on-ship.

The traditional practice of a ROV navigator at sea was maintained and the addition of a navigator onshore was drawn from Cruise A data on scientists’ understanding and requests from shore to the ship. The navigator that was co-located with team onshore for the first few days of Cruise B assisted and trained the scientists in operational matters, including the preparation of basemaps and waypoints and ROV dive logistics, such that upon their departure a subset of scientists carried lessons learned into their enriched communication.

Cruise B was separated into two distinct work modes, for the first forty percent of actual ship-time, scientists onshore did not have the ability to direct ROV dives over audio channels or via online chat. Instead, the team onshore and on-ship relied on the use of a communication tool designed for the purpose of enabling the team onboard to carry out onshore scientists’ directions as written, while being observed by scientists onshore who would provide further direction after assessing the outcome of the dive. This imposed latency between ship and shore communication met a proposed condition normative for space exploration.

SUBSEA’s “Dive Recovery and Data Report” was a multi-page document that was reflexively developed based on Cruise A work practices and SUBSEA goals. Written exchanges between ship and shore were made twice within a 24-h period. In the latter sixty percent of the Cruise B viable dive period, communication restrictions were lifted and leading dives from shore was possible. Data showed that the onshore team did not resume normative practice of individual communication via phone or text, group use of online chat or email. Except for an unplanned increase in dive time called for by the chief scientist onshore, scientists continued with prepared dive plans and to operate along earlier conditions. The earlier dives established that remote science was supportable using: 1) the practice of a written robust yet critical information-only focused document, 2) a daily telecon during which the distributed team used the shared document as an agenda, and 3) focused efforts from the ship to actively provide situational awareness details onshore which would normally be acquired on-ship, e.g., Captain updates and conversations. Cruise B objectives for all three pillars were met (Lim et al., 2021) (ocean science publications include Chan et al., 2023; Milesi et al., 2021).

The 2018–2019 SUBSEA project was the first study conducted to examine an experienced and repeat team of scientists across the conditions of telepresence-enabled and remote science. For the ocean science team, the difference in these two conditions was in large part matters of on-going real-time two-way communication and distributed group decision-making. These were integrated into the already well-known ocean science at-sea capabilities of time-sensitive decision-making, data management, and use of telecommunications. SUBSEA’s Operations pillar was able to support this integration drawing from their work with remote science models for space exploration missions and research. The SUBSEA expeditions illustrate several differences between telepresence and remote science, while the 2020 West Coast National Marine Sanctuary expeditions (Section 2.3.3) provide insights into the use of remote science by researchers who intended to be at sea. Both sets of expeditions allow the development of tools and practices to guide future at-sea science conducted remotely.

#### 2.3.3 Applied use of telepresence: west coast National Marine Sanctuary expeditions (2020)

Although telepresence technologies had supported scientific expeditions for over a decade, the 2020 COVID-19 pandemic pushed the research community to trade the at-sea experience for remote science due to health and safety concerns (e.g., Beaman, 2020; Raineault et al., 2021). The following is a review of three telepresence-enabled E/V *Nautilus* expeditions that took place between 20 September–26 October 2020.

While the vessel schedule and science teams were largely formed by late 2019 for expeditions occurring in 2020, researchers had roughly three months to pivot from their plans to be physically at-sea to remote science expeditions. It was a coincidence that these plans had the benefit of lessons learned on remote science at sea as recently as 2019. This change necessitated additional advance preparation and communications to ensure the participants understood objectives, protocols, and tools to conduct their remote work. The need to share information and coordinate with disparate individual scientists helped reinforce tools and workflows in advance of the expeditions. It also opened the door for greater inclusion of research objectives and personnel.

Pre-expedition time was spent in community or smaller team planning calls, writing an expedition plan that included a broader range of participating scientists to help co-lead ROV dives, and creating a community specimen request list and accompanying protocols for sampling and shipboard sample preservation. The specimen requests were coordinated across west coast National Marine Sanctuary sites (Olympic Coast, Greater Farallones, Monterey Bay, and the Channel Islands), which led to a better understanding of the sample needs, including the addition of photographs to aid non-experts in identification. Dive plans were created in an online shared document, which also increased access and visibility to the wider science team for input. Daily phone calls supported the shore and ship leads to connect to share progress, understand limitations, and to prioritize operations. The addition of a new audio communications software increased the number of participants onshore who could join in to provide input during dives. It also meant that dives that might not have been considered under normal conditions, such as an assessment of the commercially important Petrale sole habitat off California, was prioritized because those experts could lead the dive from shore but would not otherwise be on board the vessel. Another example was the addition of a dive to take additional samples at the site of a potential meteor impact led by NASA scientists in the Olympic Coast National Marine Sanctuary, in which five experts joined remotely.

After the expeditions, scientists gave feedback that included praise for the ability to include more experts, including outreach experts to discuss the importance of the exploration and the relevance to the public, who view and listen to the same video/audio streams on a website. In addition to outreach specialists, local groups not often included were enabled to participate in the dives and to provide knowledge and historical context. The lead scientists of each expedition reported no reduction in scientific objectives due to the remote science conditions. Twenty-six ROV dives were led primarily from shore with a rotating team of 30 watch co-leads, while three data loggers onboard the vessel assisted on watch and with sample preparation. This is a large increase in the number of experts who verbally directed dives (typically there are three watch leads per expedition). Goals for both visual transects and sampling were met with 393 samples (754 with subsamples) collected over the three expeditions.

With an entirely distributed group of co-lead scientists, there were added challenges to conducting remote science. First, scheduling watch leads who are shore-based is complicated by the dynamic ship schedule. Delays due to weather, equipment failures, resource, personnel, and other issues are traditionally managed on-ship in relation to only the ship, which continues to operate as a total institution (see Zurcher 1965 for an account of how shipboard organization meets sociologist Erving Goffman’s (1961) criteria). However, these schedule changes can cause conflicts with onshore work, home, and life-routine schedules. This is particularly problematic if a planned dive requires specific expertise. Indeed, on-ship assignments are valued in part because of the single-focused set of schedule conflicts and work expectations. Other benefits are the life-routines that exist on-ship, such as three prepared meals daily, no commute, lack of caregiving responsibilities, reduced/limited expectations for non-expedition related work. These conditions are not extended to a distributed (or often even a remote) work environment onshore. Technical requirements are also the responsibility of individuals including procuring computer(s), monitor(s), audio equipment, configuring Virtual Private Networks (VPNs) or other software needed to participate, and a reliable, high-speed internet connection. Finally, there is a greater need for advance and additional communication and preparation to work with distributed teams. Additional tasks might include writing or filming protocols for technicians/operators at-sea, objectives-driven detailed dive plan writing, and contingency plans in the event of technical failures (e.g., the loss of satellite connection to the vessel). Overall, more time is required in advance of an expedition to ensure remote science success. Communications during an expedition are also more formal—shipboard meetings over meals or in the lab to discuss progress and plans must now be communicated either in writing, verbally or both with a remote team.

## 3 Discussion

### 3.1 Defining remote science at sea

Remote science at sea is an ocean science expedition with four key components that are social, spatial, technical, temporal: 1. It is sea-going ocean science primarily led by scientists who are either co-leading from shore and ship or leading in real-time from shore. 2. Each location, both on-ship and onshore, has telecommunication, networking, and computer technologies to support the social interaction among the geographically separated lead scientists. 3. Temporally, communication exchanges are in real-time, i.e., no latency, or near-real-time, i.e., low-latency. 4. The real-time aspect is not a “seat of the pants” approach; it is enabled by requisite pre-planning for shared tools that includes expedition plans, science objectives, expectations of ROV configuration for sampling and instrument use, and communication plans.

The configuration of a workgroup geographically separated but tightly coupled to produce a shared goal, outside of ocean sciences, is described as a distributed workgroup (Baba, 2001). Within a distributed workgroup, the allocation of decision-making power is determined by the structure of the organization and social habits of the professional community. In comparison to distributed workgroups and telepresence, the distinguishing social and technical features of remote science at sea are that decision-making power is shared by participants without uniformly defaulting to onboard leadership and the use of telecommunication for real-time two-way communication. The exceptions, as always, are made by the research vessel capabilities and its crew, the ROV and operators, and the physical environment.

The authors put forward that each of these elements is part of remote science at sea and an expedition that incorporates a modicum of components is not remote science. Telepresence-enabled science is aided by the technologies but does not require remote participant support for successful completion of objectives. Shoreside persons might view live streams of video and data for education, entertainment or research purposes, but their participation is unplanned or not required. Remote science engages a team of scientists throughout the planning through publication phases of the project, with the expectation that some persons will remain onshore. These shoreside participants provide leadership while the vessel is at sea. There are no data (or reasoning found by the authors) to suggest remote science at sea upends or is meant to wholly replace either telepresence-enabled education, outreach, and ocean science or sea-going ocean science.

Widespread adoption of remote science at sea is slow to develop for institutional reasons: professional culture, structural support, and funding. Professional researchers, by the very definition of their training, are steeped in a traditional culture that values the physicality of being at sea in-person over that of being onshore, including hands-on data and sample collection, (e.g., their own eyes or hands or that of an entrusted student or colleague). Indeed, the at-sea experience is a noted reason that many choose this profession. Over a hundred years of ocean science work practice was not radically changed with the introduction of telepresence for ocean research. Seafaring is part of the profession’s identity that cannot be easily shed (e.g., Deacon, 1971; Helmreich, 2009; Rozwadowski, 2008). It is identity that drew people to this field of work, whether for the love of the sea, ocean science, marine life, or environmental stewardship (Carson, 1951). Going to sea is itself a reward for many in the ocean science research community, whether or not they are able to go to sea (e.g., interest alone is not enough, funding and schedules still matter). Another two rewards–professional accomplishment and community recognition–contribute (though not yet equally) to the community granting value to telepresence-enabled outreach and education, and research and remote science at sea.

For seagoing researchers, professional progress can be stalled by the inability to gain access to the sea. Not all people interested in or already working in ocean-faring research are able to gain support to be at sea or are able to go to sea. There is a known attrition of ocean scientists, particularly females during childrearing and elder-care years and this is a huge loss of talent during some of the most productive years of a person’s career (National Academies of Sciences Engineering and Medicine, 2024), resulting in fewer contributions, leaders, and role models from groups with caregiving responsibilities. Additionally, there are groups of persons for whom an ocean science career seems impossible—those who cannot physically go to sea. A loss of potential contributors from the outset will only be overcome once marine science is seen as a possibility through the development and acceptance of work practices like remote science. What the 2020 expeditions showed, is that given the choice of not being able to conduct any science or needing to utilize remote science to conduct an expedition, scientists prefer and can successfully adopt those practices. In addition, these expeditions demonstrated some surprising benefits over the traditional models of an entirely at-sea science party.

Increased expertise provided by onshore researchers led to a wider range of science objectives being met. For example, on SUBSEA the real-time data from the ROV dives was used by a geochemical modeler who ran simulations and provided input for future dives (Milesi et al., 2021). Some cruises can include additional fields of study through remote science. For example, during the 2020 NMS expeditions a Petrale sole spawning habitat site was characterized and surveyed and a meteorite impact site investigated because the experts could help lead the dive from shore. Since these were niche objectives, these experts likely would not have had a spot on the vessel and therefore could only be included via remote science. Similarly, maritime historians have been early adopters of telepresence to conduct remote science at-sea since surveys of maritime historic sites are often not primary goals of expeditions but can be accommodated when expedition objectives are nearby (e.g., Ballard, 2004; Brennan et al., 2018; Coleman et al., 2011; Delgado et al., 2018; Lobell, 2024; Malakoff, 2019). The historians, archeologists, and other experts actively plan, write permit applications, and lead the dives, although they do not sail on the ship, helping to address important historic and environmental questions (e.g., Ballard et al., 2018; Brennan, 2024). These examples demonstrate that a range of integration with the overall expedition objectives can be met with telepresence tools, ranging from scientist(s) aiding in the planning and execution of a single or few dives within an expedition due to their expertise to scientist(s) being involved in an entire expedition’s mission objectives. Many of the technological and social work needs are the same, although much can vary on an individual basis in terms of requirements for data and communications tempo and tools.

Structural support for remote science at sea takes into account both shipboard and shoreside requirements. To successfully conduct remote science, technologies must be in place on board the vessel and at the remote location(s) to enable real-time communications and video and data viewing. Some of the structural support can be provided through funded facilities (e.g., research vessels, deep-sea robotic assets, remote hub sites like the Inner Space Center), but some must be resourced by the individual researcher(s) through proposals. To do this, a researcher needs to know what is provided through a facility operator and what should be added to a proposal. Additionally, scientists directing and utilizing the capabilities must be present and able to focus on the expedition (remote science is no more a silver bullet for change than any other technology). Shore-based leads need to block their expedition schedule from other shoreside activities as much as possible and convey to colleagues that they are “on an expedition” to protect the time. Meeting at a shore-based hub can help ease the aforementioned types of structural issues through providing space equipped for remote science with on-staff experts and by providing a space to gather multiple remote scientists away from home institutions and routines.

Over time, structural support for remote science at sea has become more robust due to the development and use of technologies including satellites with higher and more flexible bandwidth, improved networking, increased data storage and teleconferencing tools, etc. However, along with viewing this direction, as some will, as a timeline of technological progress, these technologies can be understood as a collection with different affordances, constraints, and costs, which can be evaluated for use by individual research team’s requirements. For example, the selection of an observation or event logging system by a vessel or facility will have certain costs (e.g., monetary, time) and limitations (e.g., number and locations of users, availability of the interface after an expedition) (Cantwell et al., 2020). Data visibility on shore provides scientific and situational tools for decision-making and this can be accommodated multiple ways depending on the requirements (e.g., screens shared ashore versus live graphical displays and/or data files). Importantly, the use of multiple communications tools to improve remote work collaborations have become familiar in the post-pandemic period, and a need to codify their use during remote science at sea expeditions helps to create a record and share information with persons in different roles and locations. Immediate communication of information via text-based chat or verbally allows decision-making quickly, while written documents such as ship-shore reports of activities (e.g., “Situation Report,” “Dive Recovery and Data Report”) and dive plans allow multiple persons to create a plan or report that becomes part of the scientific record. Daily planning calls can also ensure teams understand mission plans and constraints. With all these additional considerations, there is recognition that the work of planning, coordinating, and conducting an expedition involving remote science at sea often requires additional personnel and/or time from knowledgeable persons (Cantwell et al., 2020). The academic research community can continue to learn best practices through trial and error (e.g., Kaiser et al., 2012; Dalpe et al., 2022) and through public-private partnerships. Industries producing technology for commercial ocean interests have contributed to the development of vehicle systems and telepresence technologies (e.g., Matthews, 1981; Chamberland, 1987). Publicly funded scientists have outlined practices to collect data of scientific value using industry ROVs (McLean et al., 2020). Public-private partnerships may further be developed as opportunities, including to extend the reach of ocean science data collection, application of analysis, training of a blue workforce, and innovations in data management.

Vessels need to be equipped with satellite(s), networking, communication, and data management technologies to support remote science. Mobile telepresence units are portable and have supported telepresence mainly for education and outreach aboard vessels but are temporary and have integration costs. The ISC noticeably supports projects reviewed in this article, but this was not by design (in other words, the authors did not select projects to review based on whether or not a project utilized the ISC). There are financial and social benefits to co-locating a shore team at a single location or a few shore locations that have the infrastructure in place to support remote science. While researchers can set up their own shoreside remote science “center,” this requires technical expertise, their own set-up and support of stable local infrastructure. The Exploration Command Centers at locations onshore to support telepresence-enabled research vessels have largely been replaced (or no longer setup by earlier designs) with the now more common communication technologies (e.g., video conferencing/VOIP for communication, web-based live data visualization and event logging tools).

Until conducting remote science at sea using telepresence technologies becomes a widely adopted practice, opportunities to train ocean scientists and vessel and robotic operators will need to be defined, scoped, and funded. Developing an understanding of the opportunities, needs, and challenges with attention from multiple disciplines will help grow practices and imbue new values. Encouraging discussion of these through professional meetings, workshops, and conferences, as well as in publications, can also help develop a community of practice for remotescience at sea.

## 4 Conclusion

Remote science at sea is now an option for persons to conduct sea-going research. While this review focuses on use for ROV-aided research, the remote science configurations for ocean scientists doing other types of marine research can also be developed. The technological advances over the last twenty plus years have lowered costs and made it possible for persons onshore to actively lead and participate in expeditions. And yet, support and effort are needed to aid researchers in expedition planning, managing increased participation (humans and technology), requesting funds, sample and data management, and public communication.

The growth of productive ocean expeditions with remote science and telecommunication technologies is met with social, technical, and logistical challenges. Authors can offer some direction for remote science expeditions on research vessels conducting science with distributed teams at sea and onshore. Pre-expedition planning through both face-to-face interaction and written documents helps to familiarize and establish communication practices that reflect the particular team. Even with shared membership in the professional community of ocean science, subgroups (or subcultures) increase familiar communication and adopt shared values through their shared practices. Group dexterity, developed through actual use, with telepresence communications systems will allow communication mediums to become recessed (e.g., normal infrastructure). Data management planning in advance of data collection needs to include two temporal spaces: real-time data collection ascertained against the planned science objectives and post-expedition distribution. Distributed teams should be equipped with technology that supports visualization of data and situational awareness (e.g., maps, people, places).

As more research vessels are equipped with high-bandwidth satellite capabilities for constant, low-latency connectivity to shore, additional infrastructure is needed to assist remote science such as vessel network infrastructure to prioritize science use of satellite bandwidth, robust data management systems to allow remote data access and communications equipment to have integrated audio-visual communications. These technical considerations are standard on telepresence-enabled vessels but need to be planned for and provisioned on vessels of opportunity. Technical support to ensure data synchronization, live-streaming, and communications capabilities may require additional support or training for technicians on board vessels. Integrated communications between ship and shore might include accommodations for instant communications (e.g., text messaging, chat), cruise and/or dive event logging, scheduled written and verbal daily briefs and plans, and on watch or between watch calls or videoconferencing.

Scientists need to be familiarized with the capabilities and best practices of remote science and have a clear pathway to request the capabilities through funding agencies. Both changes will help the community see remote science as a viable option for science, not just broader impacts for outreach and education. To do this, the cost of remote science must be better understood. Providing remote science options during the grant proposal process as a facility cost would help reduce the burden for researchers to determine what and how to budget for these expenses.

Some of the skills that successful remote science requires includes attention to detailed pre-planning and communication. The lead scientist must consider how much science can be accommodated, as there are resource limitations such as sample space on a vehicle, total dive or expedition time, persons on board to handle samples, vehicle/ship capacity for additional sensors or equipment, data and sensor management. During the expedition the lead(s) need to focus on the range of objectives and proceed methodically to achieve multiple goals. Decision-making based on clearly communicated objectives in expedition and dive plans helps watchstanders, who may need to make decisions for a teammate, such as in the event of a satellite outage. Guidance developed as standard operating procedures for routine operations or sample, or instrument handling can also ease communications and workflows.

Additional opportunities to develop remote science skills (both as a scientist at-sea and onshore) can build a community equally equipped to conduct science onboard a vessel and from shore. Chief scientist training programs, ocean research internships, and other seagoing marine science professional development opportunities can offer this as part of their programming to build understanding and acceptance as a practice. Senior scientists have a significant role in building acceptance through the ways in which they teach, develop, and reward students and colleagues. Indeed, as authors state earlier, it is worth challenging descriptions that equate the activity of participating from onshore with watching a movie, as some scientists have referred to as a “get some popcorn” activity, when in fact on-ship scientists have always used video monitors to view ROVs operating undersea from a distance.
